# Quality Attributes, Structural Characteristics, and Functional Properties of Brewer’s Spent Grain Protein Concentrates as Affected by Alkaline and Pulsed Electric Field-Assisted Extraction

**DOI:** 10.3390/foods14091515

**Published:** 2025-04-26

**Authors:** Parichat Paksin, Pipat Tangjaidee, Wannaporn Klangpetch, Kridsada Unban, Tabkrich Khumsap, Warinporn Klunklin, Artit Yawootti, Kittisak Jantanasakulwong, Pornchai Rachtanapun, Suphat Phongthai

**Affiliations:** 1 Faculty of Agro-Industry, Chiang Mai University, Chiang Mai 50100, Thailand; parichat_paksin@cmu.ac.th (P.P.); pipat.t@cmu.ac.th (P.T.); wannaporn.u@cmu.ac.th (W.K.); kridsada.u@cmu.ac.th (K.U.); tabkrich.khumsap@cmu.ac.th (T.K.); warinporn.k@cmu.ac.th (W.K.); kittisak.jan@cmu.ac.th (K.J.); pornchai.r@cmu.ac.th (P.R.); 2 Center of Excellence in Agro Bio-Circular-Green Industry (Agro BCG), Faculty of Agro-Industry, Chiang Mai University, Chiang Mai 50100, Thailand; 3 Department of Electrical Engineering, Faculty of Engineering, Rajamangala University of Technology Lanna, Chiang Mai 50300, Thailand; yartit@rmutl.ac.th; 4 Lanna Rice Research Center, Chiang Mai University, Chiang Mai 50100, Thailand

**Keywords:** pulsed electric field, brewer’s spent grain, functional properties, alternative protein

## Abstract

The rising protein demand has driven intensified research into alternative protein sources and extraction technologies. Brewer’s spent grain (BSG), which is rich in protein, remains mostly underutilized. This study aimed to optimize BSG protein extraction conditions using pulsed electric field (PEF) by assessing the influence of pulse numbers (5000–9000), electric field strength (8–10 kV/cm), and frequency (8–10 Hz) on protein recovery and purity. The optimized conditions (5386 pulses, 10 kV/cm field strength, and 10 Hz frequency) provided a higher extraction yield with a significant improvement of approximately 90% (*p* < 0.05). Essential amino acids in proteins extracted via PEF were significantly increased (60,864.84 mg/100 g), particularly phenylalanine, threonine, and valine; furthermore, amino acid score (AAS) and protein digestibility-corrected amino acid score (PDCAAS) were found to be superior to those of protein obtained through alkaline extraction. The PEF treatment resulted in the modification of the secondary structures of proteins from α-helices and β-turns to β-sheets, as well as the enhancement of the hydrophobic−hydrophilic amino acid balance. The functional properties of the proteins, particularly their foaming properties and solubility, were significantly affected by PEF (*p* < 0.05). In conclusion, PEF-assisted extraction produces high-quality BSG protein concentrates efficiently while rendering the process environmentally sustainable.

## 1. Introduction

The world’s population has been growing quickly and it is expected to reach over 9 billion people by 2050 [1]. Food supplies are severely strained by this fast increase, which raises the need for efficient and sustainable sources of nutrition, especially protein [2]. Research is increasingly focused on alternative protein sources that potentially reduce waste and enhance environmental sustainability [3,4].

Brewer’s spent grain (BSG), including up to 85% of total brewery waste, is a byproduct of the brewing business [5]. BSG has historically been considered an underutilized resource due to its high protein content, which constitutes roughly 25% of the overall grain composition [6]. Approximately 0.2 kg of brewer’s spent grain (BSG) is generated for each kilogram of beer brewed. Over the past three years, the brewing sector has generated in excess of 190 billion L of beer [7], resulting in the production of over 38 million tons of brewer’s spent grain, of which 9.5 million tons of protein remain unutilized. BSG offers significant potential for upcycling into value-added products, in addition to its use as animal feed [8]. The extraction of protein from BSG could lead to novel applications in the food and nutraceutical industries, contributing to the sustenance of the expanding global population and promoting more sustainable food systems.

Protein extraction is a crucial step in the value-adding process for protein-rich byproducts, such as BSG. Traditional extraction methods, including enzymatic and chemical or alkaline extraction (ALK), have been widely utilized due to their cost-effectiveness and simplicity [9]. Nonetheless, protein denaturation, inadequate selectivity, protracted processing durations, and potential environmental concerns are among the limitations of these techniques [5]. Consequently, the utilization of some advanced technologies such as microwave, ultrasound, and pulsed electric field (PEF) have been investigated to enhance the efficiency of protein extraction [10,11]. Microwave-assisted extraction (MAE) and ultrasonic-assisted extraction (UAE) utilize microwave and ultrasound energy to thermally process materials, therefore improving solvent penetration and protein release, respectively. Nonetheless, these techniques may lead to overheating, possible degradation of proteins, and inconsistent energy distribution, which could negatively affect protein quality [12,13]. Meanwhile, PEF is a nonthermal processing technique that employs a short, high-voltage electric pulse to permeabilize cell membranes, facilitating efficient protein release with minimal thermal effects [14].

The principle of electric field strength, utilized in PEF-assisted extraction, is conveyed in short pulses between two closely positioned electrodes. The electric field intensity generated between the electrodes will lead to a potential difference across the cell wall, which in turn creates holes in the cell membrane and enhances the permeability of the wall. The term for this phenomenon is “electroporation” [15]. The phenomenon arises when the intensity of the electric field surpasses the critical threshold, resulting from a significant buildup of electric charge within the cell membrane. The porosity of the cell membrane leads to an increased mass transfer rate of compounds between the interior and exterior of food cells [16]. As a result, this promising principle has garnered attention for its potential to improve the efficiency of protein extraction. Additionally, alterations in the protein’s secondary structures have been discovered, leading to a notable improvement in foaming, emulsification, and oil-binding capacity [17,18].

The literature review suggests that malt residue from beer production contains protein levels that are adequate to serve as valuable food components. Furthermore, the utilization of pulsed electric field technology to improve the efficiency of protein extraction from BSG is still constrained. Consequently, the objective of this investigation was to determine the most suitable conditions for the extraction of protein concentrate from BSG using PEF-assisted extraction. The PEF process was compared to the traditional alkaline method in terms of extraction yield, changes in protein secondary structure, and protein quality parameters, including essential amino acid ratio, amino acid score (AAS), in vitro protein digestibility, and protein digestibility-corrected amino acid score (PDCAAS), as well as functional properties.

## 2. Materials and Methods

### 2.1. Raw Material Preparation

Brewer’s spent grain (cv. Samoeng 2 barley) was obtained from the production of non-alcoholic beer using the conditions reported in [19]. BSG was subjected to drying at 55 °C in a hot air oven (Memmert GmbH + Co. KG, Schwabach, Germany) for 16 h (moisture content < 10%). The dried BSG was subjected to mashing and subsequently sieved using a 100-mesh sieve. The sample powder was kept in an aluminum foil bag at −18 °C for subsequent experiments.

### 2.2. Alkaline Extraction

BSG powder was dispersed in distilled water (1:10 *w*/*v*), followed by pH adjustment to 10 using 1 M NaOH. The mixtures were stirred at 50 °C for 90 min with an overhead stirrer (IKA RW 20 Digital, Wilmington, NC, USA). After centrifuging at 5500× *g* at 4 °C for 15 min (Himac CR 22N, Germany), the top fraction was collected, and the pH was adjusted to 4.5 using 1 M HCl. The protein pellet was recovered via the same centrifugation condition. The protein precipitate was neutralized and freeze-dried (LABCONCO 25L, Kansas City, MO, USA) to obtain protein concentrate (PC).

### 2.3. Pulsed Electric Field-Assisted Extraction

The experimental design was created using Design−Expert version 6.0.2 (Stat-Ease, Minneapolis, MN, USA). A three-level, three-factor Box−Behnken design along with response surface methodology (RSM) was employed to assess the effects of the number of pulses (X_1_, 5000−9000 pulses), electric field strength (X_2_, 8−10 kV/cm), and frequency (X_3_, 8−10 Hz) on protein recovery and purity. The experimental design comprised 15 treatments with three replications of the center point, as shown in Table 1. The PEF treatments were performed using a bench-scale pulsed electric field apparatus in batches using exponential decay pulses (1 μs). A probe electrode (2.0 cm diameter) was inserted into a cylinder chamber (6.0 cm diameter, 24.0 cm height) containing 200 mL of the sample mixture (1:10 *w*/*v*, pH 10) and an overhead stirrer was used to continue stirring the solutions for 90 min at 50 °C after they finished PEF treatment. The protein precipitation step was applied to the mixture of each treatment, as described in the section on ALK. These samples were used to determine the best protein purity and recovery. The purity of the protein indicates the protein content in the extracted sample, which was quantified following the methodology outlined in Section 2.4. Meanwhile, protein recovery was calculated using the following equation:

$$\mathrm{Protein}\;\mathrm{recovery}\;(\%)=\frac{\left[\left(\%\mathrm{Protein}\;\mathrm{in}\;\mathrm{BSG}\;\times \;\mathrm{Weight}\;\mathrm{of}\;\mathrm{BSG}\;\mathrm{used}\right)-\left(\%\mathrm{Protein}\;\mathrm{in}\;\mathrm{PC}\;\times \;\mathrm{Weight}\;\mathrm{of}\;\mathrm{PC}\;\mathrm{derived}\right)\right]\times 100}{\left(\%\mathrm{Protein}\;\mathrm{in}\;\mathrm{BSG}\;\times \;\mathrm{Weight}\;\mathrm{of}\;\mathrm{BSG}\;\mathrm{used}\right)\;}$$

A regression coefficient was derived by fitting the experimental data to a quadratic polynomial model. In the response surface analysis, the following generalized quadratic model was employed:

$${Y=\beta }_{0}+\sum_{i=1}^{3}\beta \mathrm{iXi}+\sum_{i=1}^{3}{\beta \mathrm{iiXi}}^{2}+\sum_{i=1}^{2}\sum_{i=2}^{3}\beta \mathrm{ijXiXj}$$

where β_0_ is the constant, β_*i*_ is the linear coefficient, β_i*i*_ is the quadratic coefficient, and β_*ij*_ is the interaction coefficient. *X*_*i*_ and *X*_*j*_ are the levels of the independent variables.

The effect and regression coefficients of individual linear, quadratic, and interaction terms were estimated and generated in the analysis of variance (ANOVA), Table 2. The significance of all terms in the polynomial was assessed statistically by computing the F-value at a probability (*p*) of 0.05. The three-dimensional contour plots from the regression models were produced using statistical computations based on the regression coefficients.

### 2.4. Proximate Analysis

The moisture, protein, fat, and ash content of protein powders obtained from ALK and PEF treatments were analyzed utilizing the AOAC (2000) method, specifically methods 927.05, 984.13 (*N* × 5.95), 942.05, and 920.39B, respectively.

### 2.5. Protein Quality Determination

#### 2.5.1. In Vitro Gastrointestinal Digestion

In vitro gastrointestinal digestion using pepsin–trypsin, as outlined by [20], was employed to assess the digestibility of PCs. Pepsin (enzyme/protein ratio of 1:100, *w*/*w*) was incorporated into the PC dispersions (pH 1.5, 1%, *w*/*v*), and the mixture was gently agitated at 37 °C for 2 h. The enzyme activity was then stopped by neutralizing the mixture with 3 M NaOH. Subsequently, trypsin was introduced to the previously digested mixture at an enzyme-to-protein ratio of 1:100 (*w*/*w*). Following 2 h of the second digestion at 37 °C, the trypsin activity was terminated by heating the mixture to 95 °C for 10 min, after which TCA was added to an equivalent volume of samples. Following the centrifugation of the solutions for 10 min at 5500× *g*, the sediments were collected and subjected to freeze-drying. The protein content was subsequently analyzed and the formula outlined below was utilized to assess protein digestibility:

Protein digestibility (%) = (A − B)/A × 100

where A is the protein content in the digested protein sample and B is the protein content in the initial protein sample.

#### 2.5.2. Amino Acid Profile Determination

The amino acid profile was determined using the amino acid analyzer, as outlined by [21]. The protein samples were digested with 6 M HCl at 110 °C for 24 h. The solution was then diluted using sodium citrate buffer, and the pH was adjusted to 2.2. Amino acids in each sample were separated using a Zebron ZB-AAA capillary GC column (0.25 mm i.d. × 10 mm with film thickness of 0.25 µm) and analyzed via GC–MS (6890N; Agilent Technologies, Santa Clara, CA, USA). The quantity of each amino acid was expressed in milligrams per 100 g of protein.

#### 2.5.3. Amino Acid Score Determination

Amino acid score (AAS) is a recognized indicator of protein quality, having been defined and endorsed by FAO expert groups. AASs are designed to reflect the capacity of dietary protein to fulfill amino acid requirements, as outlined by the FAO/WHO/UNU Expert Consultation in 1985. The equation used to calculate the AAS is as follows:

$$\mathrm{AAS}=\frac{\mathrm{Milligram}\;\mathrm{of}\;\mathrm{essential}\;\mathrm{AA}\;\mathrm{in}\;1\;g\;\mathrm{of}\;\mathrm{test}\;\mathrm{protein}}{\mathrm{Milligram}\;\mathrm{of}\;\mathrm{the}\;\mathrm{same}\;\mathrm{AA}\;\mathrm{in}\;\mathrm{requirement}\;\mathrm{pattern}}$$

The essential amino acid that is least adequate in fulfilling dietary needs is referred to as the limiting amino acid.

#### 2.5.4. Protein Digestibility-Corrected Amino Acid Score Determination

Protein digestibility-corrected amino acid score (PDCAAS) serves as an indicator for evaluating protein quality through the examination of amino acid score of limiting amino acids and digestibility.

PDCAAS (%) = Amino acid score of limiting amino acids × Protein digestibility (%)

### 2.6. Determination of Protein Secondary Structure Changes

According to [17], an FTIR spectrometer (Tensor 27, Bruker, Ettlingen, Germany) was used to investigate the transmission infrared spectra of each protein sample. After being combined with KBr, the samples were compacted into a pellet. The measurement was carried out in the range of 400–4000 cm^−1^ with a resolution of 4 cm^−1^. The OriginPro 2022 program (OriginLab Corporation, Northampton, MA, USA) was utilized to separate the amide I region spectra (1600–1700 cm^−1^), into multi-component peaks including β-sheets (1611 and 1626 cm^−1^), random coils (1642 cm^−1^), β-helices (1657 cm^−1^), and β-turns (1673 and 1688 cm^−1^).

### 2.7. Functional Properties Determinations

ALK- and PEF-treated PCs were assessed for functional properties, including solubility, foaming and emulsifying properties, and oil-holding capacity, using the modified methodology of [20].

#### 2.7.1. Solubility

The sample was dissolved in distilled water in 1% (*w*/*v*) and pH was adjusted to 3, 5, 7, 9, and 11 using 1N HCl or 1N NaOH. The samples were subjected to magnetic stirring for 30 min at room temperature, followed by centrifugation at 5500× *g* for 15 min. The supernatant’s protein content was determined using the Bradford method, with bovine serum albumin (BSA) used as the standard. Protein solubility was calculated using the following equation:

$$\mathrm{Protein}\;\mathrm{solubility}\;(\%)=\frac{\mathrm{Soluble}\;\mathrm{protein}}{\mathrm{Total}\;\mathrm{protein}}\text{×}100$$

#### 2.7.2. Foaming Properties

The protein samples (1% *w*/*v*) were dissolved in 20 mL distilled water before being transferred to a plastic cylinder. Total volume was recorded at 0 and 30 min following the whipping process using a homogenizer (10,000 rpm for 1 min). Foaming activity (FA) and foam stability (FS) were calculated using the following equations:

Foaming activity (%) = [(A − B)/B] × 100

Foaming stability (%) = [(A_30 min_ − B)/(A_0 min_ − B)] × 100

where A represents the volume after whipping (mL), while B denotes the initial volume (mL).

#### 2.7.3. Emulsifying Properties

The protein samples (1% *w*/*v*) were dissolved in 10 mL distilled water before mixing with 10 mL of soybean oil. The emulsion was subsequently prepared by homogenizing at 10,000 rpm for 1 min. The emulsifying activity (EA) and stability (ES) were estimated using the equations outlined below:

Emulsifying activity (%) = [(A/B)] × 100

Emulsifying stability (%) = [(A_incubate_/A)] × 100

where A is the emulsified layer volume, A_incubate_ is the volume after 10 min at 80 °C, and B is the total volume.

#### 2.7.4. Oil-Holding Capacity

After mixing 0.5 g of protein samples with 5.0 mL of soybean oil, the mixture was centrifuged for 15 min at 1200× *g*. After removing the top fraction, the oil-absorbed fraction was weighed, and the oil-holding capacity was calculated using the following formula:

Oil-holding capacity (g/g) = Weight of absorbed oil (g)/Weight of sample (g)

### 2.8. Statistical Analysis

Design Expert^®^ Software (version 6.0.2) was employed for the optimization of experimental data. A one-way analysis of variance (ANOVA) was performed, followed by Duncan’s multiple range test, to compare the means among the groups utilizing SPSS Statistics version 17.0 (SPSS Inc., Chicago, IL, USA).

## 3. Results and Discussion

### 3.1. Effect of PEF on Protein Extraction

#### 3.1.1. Model Fitting and Statistical Analysis

A Box–Behnken design with three repetitions at the center point was used in the experiment to determine the optimal conditions for PEF-assisted extraction of protein using the response surface methodology. This study included 15 experimental runs in total (Table 1). Number of pulses, electric field strength, and frequency were the three factors that were examined at three different levels to determine two response values: the protein recovery and the purity of protein concentrate. As a result, protein recovery and purity ranged from 3.54% to 25.48%, and 75.62% to 91.60%, respectively.

A multiple regression model at a significance level of α = 0.05 (Table 2) was utilized to examine the relationship between the three factors influencing protein purity and the recovery of concentrated protein from BSG. It was discovered that the equation predicting concentrated protein purity had a probability value (*p*-value) of 0.0004. This suggested that the experimental values correspond to the model’s predictions (*p* < 0.05). According to the coefficient of determination, which was 0.9861, the equation has a 98.61% prediction accuracy. At the 95% confidence level, the model’s inadequacy is not statistically different from the experimental results, as evidenced by the model’s lack of fit. As a result, a quadratic model can use the equation.

On the other hand, the protein recovery prediction equation had a probability value of 0.8362, which shows that the predictions and experimental values are different. Furthermore, the prediction accuracy of the multiple regression equation for this response variable was only 46.67%. The experimental results and the model’s lack of fit revealed a statistically significant difference at the 95% confidence level. Therefore, the protein purity was selected as the only response variable for additional statistical analysis.

#### 3.1.2. Relationship Between Studied Parameters and Protein Purity

The relationship between the parameters being studied and the protein purity was demonstrated by each factor of protein purity response as the following regression equation:

Y = 77.64−6.22 × _1_ + 1.01X_2_ + 0.64X_3_ − 2.31X_1_X_2_ + 0.73X_1_X_3_−0.65X_2_X_3_ + 4.23X_1_^2^ + 1.04X_2_^2^ +1.65X_3_^2^

The number of pulses and the protein’s purity were found to be negatively correlated by the regression model analysis. The more pulses there were at the same electric field intensity and frequency, the lower the protein purity. Nonetheless, positive correlations between the response value and frequency and the field strength were found, suggesting that the protein purity increased in combination with these two variables. These results indicated that rapid permeation of internal compounds can result from high potential voltage because it can alter the permeability of the cell membrane or irreversibly damage the cell wall and membrane structure [10]. Ref. [22] indicated that protein extraction was enhanced by increasing the pulse number from 2 to 8, but yields decreased when the pulse number increased from 8 to 12 because of irreversible changes to proteins. Additionally, PEF may be used in biotechnological processes consisting of protein purification and extraction, where enhanced antioxidant and functional properties are intended [23].

**Figure 1:**
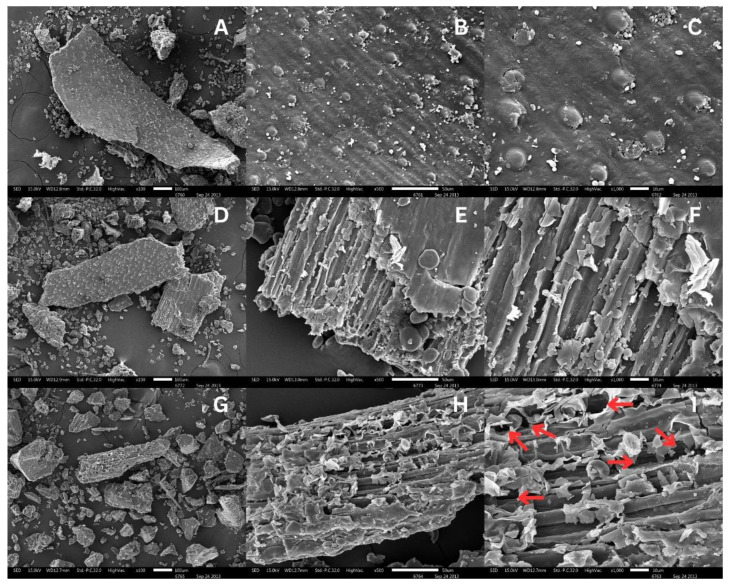
SEM images showing BSG characteristics with different treatments: untreated sample (**A**) 100×, (**B**) 500× and (**C**) 1000×; ALK-treated sample (**D**) 100×, (**E**) 500×, and (**F**) 1000×; and PEF-treated sample (**G**) 100×, (**H**) 500×, and (**I**) 1000×. The red arrows indicated the PEF-induced pores.

Using a scanning electron microscope at magnifications of 100, 500, and 1000 times, the morphology analysis of BSG revealed that the starting raw material’s surface area was comparatively smooth and devoid of porosity. On the other hand, the BSG surface that was extracted using the traditional method showed small cracks that resembled peeling from the outside, revealing the fibrous structure inside. Proteins and other constituents are moved from the inside to the outside when these fissures form. Furthermore, it is apparent that the produced electric current affects the BSG’s surface, resulting in an increased formation of pores, as opposed to extraction via traditional methods (Figure 1).

This enables the movement of compounds stored within the cells to the outside. Electroporation is a phenomenon that occurs when an electric field causes the cell membrane to gather electrical potential at the cell wall, eventually exceeding the critical value of the cell wall and leading to the creation of pores on its surface. This causes the cell wall to break or crack and to be destroyed or changed. Ref. [17] reported that the extraction of protein from rice bran via PEF-assisted extraction resulted in the formation of more pores in the cell walls, hence enhancing protein extraction efficiency. Ref. [22] investigated the influence of electric field intensity on the yield of protein recovered from mussels. The results indicated that elevating the strength from 10 to 20 kV/cm enhanced the yield of protein extraction. The yield of protein extraction augmented with strength. They attributed the efficiency of PEF to its ability to disrupt cell membranes, enabling more solvent influx and enhancing substance penetration.

#### 3.1.3. Model Validation

The predicted optimal conditions for protein extraction included 5386 pulses, a frequency of 10 Hz, and an electric field strength of 10 kV/cm. The derived condition was experimentally conducted to validate the regression model. The actual protein purity from the experiment was 89.26%, which was only 1.23% different from the predicted value when the parameters of each factor were examined. However, it was discovered that PEF can increase the protein extraction efficiency regarding protein recovery and protein purity by 1.83- and 1.22-fold, respectively, when compared to alkaline extraction alone (ALK). In addition, the optimal PEF treatment (∼9 min) required less than one-tenth the extraction time compared to ALK. Consequently, the optimal PEF condition was employed to prepare the BSG protein for subsequent comparative analysis.

### 3.2. Effect of PEF on Protein Quality

#### 3.2.1. Chemical Composition of Protein Concentrates

The chemical composition analysis revealed that PEF treatment produced a higher protein purity compared to the ALK-treated process by about 16% (Table 3). The non-thermal nature of PEF likely enhanced protein release through electroporation while minimizing denaturation and co-extraction of non-protein components. In contrast, ALK treatment resulted in higher residual impurities, such as ash and carbohydrates. Overall, PEF extraction proved superior in yielding a purer, higher-quality protein concentrate. Moreover, the protein content of the extracted concentrated protein from malt residue was found to be higher than that of the oat bran from the milling process and the walnut residue from the production of walnut oil [24,25].

#### 3.2.2. Amino Acid Profile of ALK-Treated and PEF-Treated Protein Concentrates

The influence of pulsed electric field treatment on the essential amino acid composition of protein concentrate was examined in comparison with that of ALK treatment. The results indicated that the PEF-treated sample exhibited a markedly higher total essential amino acid content (20,288.28 ± 447.24 mg/100 g) compared to the ALK-treated sample (15,293.20 ± 71.14 mg/100 g), with an increase of approximately 1.33 times (*p* < 0.05). Our results revealed that the amino acid profile of the BSG protein concentrate contained a proportion of essential amino acids of approximately 30%, which was similar to the BSG protein concentrate examined in the study of [5]. Furthermore, BSG was identified as a protein source with a greater proportion of essential amino acids than other alternative sources such as quinoa (19%) or amaranth (28%) [26].

The impact of PEF on amino acid profiles may differ based on the sources of protein and the conditions used. In addition to the induced structural conformation of proteins, PEF also resulted in increased levels of free amino acids [27]. Ref. [28] demonstrated that high-intensity PEF treatment (3 kV/cm) resulted in an increase in valine content in chicken breast protein, measuring 416.5 ng/20 μL, compared to the non-PEF-treated sample, which measured 356.0 ng/20 μL. The results indicated that protein structure is affected by increasing treatment intensity parameters, including electric field strength and the number of pulses, which may result in the release of free amino acids (FAAs). The disruption of a protein’s double layer structure by the electric field may have led to the degradation of smaller proteins or peptides into free amino acids [29,30]. However, in this study, the ratios of each amino acid present in both samples were comparable, particularly for the major essential amino acids such as valine, phenylalanine, threonine, and lysine. Therefore, the differences may be ascribed to the varying protein content of the samples rather than the influence of PEF treatment. Despite BSG protein being identified as having high purity and being a rich source of certain crucial amino acids, further evaluations are necessary to assess its compliance with requirements and digestibility.

#### 3.2.3. In Vitro Digestibility of ALK-Treated and PEF-Treated Protein Concentrates

Under simulated gastrointestinal conditions, the protein concentrate from BSG treated with ALK demonstrated a digestibility of 49.84 ± 0.10%, comparable to the digestibility of malt protein (45.6%) reported by [31]. The protein concentrate subjected to PEF treatment exhibited a digestibility of 54.96 ± 0.48%, indicating an enhancement attributed to the influence of PEF. This occurs as PEF can induce structural modifications by unfolding protein configurations, leading to an increased susceptibility of proteins to hydrolysis by gastrointestinal proteases. Furthermore, it has been observed that changes in protein structure enhance protein solubility and improve surface activity by exposing hydrophobic regions [32]. As a result, these alterations may lead to increased protein susceptibility to digestive enzymes, particularly pepsin. Ref. [33] reported that the digestibility of venison (*Cervus elaphus*) treated with PEF improved to 93.0% during in vitro digestion, compared to 91.9% for the non-treated sample. The study by [34] indicates that beef subjected to a higher voltage of 20 kV exhibited superior digestibility compared to beef treated with a lower voltage of 5 kV.

Nonetheless, various factors can influence protein digestibility, especially anti-nutritional compounds, such as phytate and trypsin inhibitors, which can impede the absorption and digestion of nutrients, including proteins [35]. This is probably a key reason why BSG protein concentrate exhibits lower digestibility. Several methods can be utilized to enhance the digestibility of proteins that are typically low in digestibility. Techniques such as cooking, high-pressure processing, and enzymatic hydrolysis can be applied to modify the protein structure, thereby increasing its accessibility to digestive enzymes. Similarly, ref. [36] found that the protein mixture of soy protein isolate and zein (5:1), when partially hydrolyzed with papain (1% *w*/*w*), exhibited a 23.9% increase in in vitro digestibility compared to unhydrolyzed protein mixtures.

#### 3.2.4. Amino Acid Score and PDCAAS of ALK-Treated and PEF-Treated Protein Concentrates

Amino acid scores (AASs) are intended to indicate the ability of dietary protein to meet amino acid requirements. The essential amino acid that is least sufficient in meeting dietary requirements is known as the limiting amino acid. According to Table 4, leucine was indicated as the first limiting essential amino acid in both the ALK-treated (0.37) and PEF-treated (0.49) samples, followed by isoleucine as a second limiting amino acid (0.53 and 0.66, respectively). Thus, it can be concluded that PEF treatment might not be sufficient to enhance the amino acid score of BSG protein concentrate.

Additionally, PDCAAS is extensively used for the evaluation of protein quality. This indicator evaluates a protein’s capacity to supply essential amino acids required for daily human consumption. The method entails quantifying the first limiting essential amino acids in a test protein as a fraction of the respective amino acids in an ideal reference form, subsequently multiplying by the protein’s digestibility [37]. According to AAS, both treatments exhibited lower PDCAASs, with values ranging from 18.44% to 24.42%. Consequently, this evidence indicates that BSG protein is not an appropriate source of amino acids, including leucine and isoleucine. Most plant-based proteins are typically regarded as incomplete proteins due to their deficiency in certain essential amino acids or insufficient levels of these amino acids to meet dietary requirements. Corn flour provides sufficient amounts of leucine and methionine; however, it is deficient in lysine, which is an essential amino acid. Soybeans exhibit a deficiency in methionine while being abundant in lysine and isoleucine [26,38]. Consequently, these protein sources may be combined in suitable ratios to produce complete protein profiles that meet human needs for essential amino acids. As the AAS improves, the PDCAASS can be correspondingly enhanced.

#### 3.2.5. Effect of PEF on Secondary Structure of Protein Concentrates

The secondary structures of the proteins in the BSG protein concentrate were examined using Fourier Transform Infrared Spectroscopy (FT-IR). The results shown in Table 3 indicate that the proportion of each secondary structure in the PEF-treated protein concentrate mostly differed from that in the ALK-treated sample. PEF significantly influenced the conversion of α-helices and β-turns to β-sheet structures (*p* < 0.05). Nevertheless, the random coils remained unchanged (*p* > 0.05).

This finding aligns with the study of [39], who found that applying PEF treatment at 10 kV to canola protein isolate alters its secondary structure, specially increasing β-sheets and random coils while decreasing α-helices and β-turns, resulting in enhanced solubility, emulsifying, foaming, and oil-holding capacity. Nonetheless, PEF has the potential to alter protein conformation in various ways, influencing changes in secondary structures. The research conducted by [40] revealed that α-helix segments transformed into β-turns or random coils under PEF conditions. This effect can be dependent upon the degree of protein folding, the hydrogen bonds formed between atoms in the polypeptide backbone, and the interactions of side chains that depend on the amino acid sequences [41]. The transition occurred almost simultaneously in two α-helix segments, illustrating the sensitivity of α-helices to conformational changes induced by an electric field.

### 3.3. Effect of PEF on Functional Properties of Protein Concentrates

#### 3.3.1. Solubility

**Figure 2:**
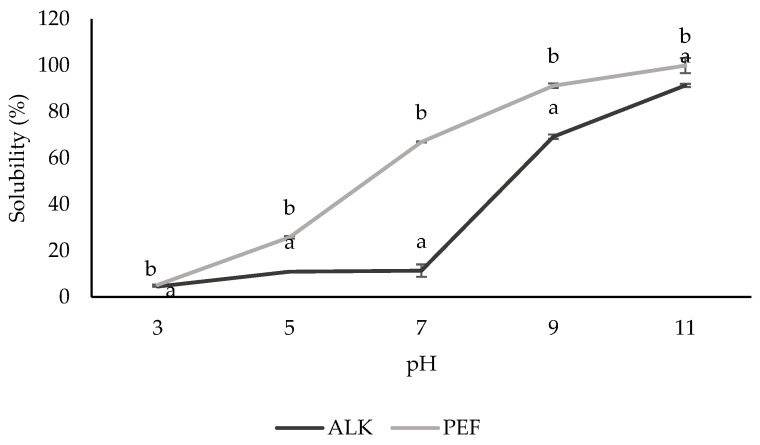
Solubility of BSG protein concentrates prepared by alkaline (ALK)- and pulse electric field (PEF)-assisted extraction. Data are expressed as mean ± SD. Different lowercase letters (a, b) indicate significant differences (*p* < 0.05).

Figure 2 shows the solubility of BSG protein concentrate prepared by ALK and PEF extraction. At pH 3, which approached the protein’s isoelectric point, precipitation occurred due to the net charge being equal to zero, thereby influencing protein precipitation and resulting in reduced solubility. However, the rise in pH affected the ionizable properties of the protein structure, hence increasing the solubility of proteins by the predominating negative charge. The accumulation of similar charges leads to repulsion among the molecules, thereby enhancing protein solubility [42].

Furthermore, it was discovered that the PEF-treated protein was significantly more soluble than the ALK-treated one (*p* < 0.05). PEF-induced structural alterations that accelerate protein unfolding and expose more hydrophilic groups are likely the cause of this increased solubility [39]. Similarly, ref. [43] found that PEF might cause the proteins in fava beans to unfold, which would probably improve their initial solubility by exposing hydrophilic areas and improving hydration. Though the exact mechanism of PEF is still unknown, the most widely accepted ideas indicate that enhanced hydration and solubility are a result of structural unfolding and peptide dipole moment polarization [44].

#### 3.3.2. Foaming Properties

The foaming properties of proteins are a vital functional attribute in food formulation, widely employed in baked and aerated products such cakes, breads, and ice cream [45]. In this study, BSG protein concentrates prepared by both ALK and PEF treatments exhibited low foaming ability. Nonetheless, PEF treatment markedly enhanced foaming ability (30.86%) and foam stability (79.37%) in contrast to ALK treatment (7.84% and 68.89%, respectively, *p* < 0.05). This finding corresponds with the research conducted by [46], which indicated that untreated chickpea protein (CP) showed a reduced foaming capacity. Furthermore, the foaming capacity of CP was markedly improved after PEF treatment (60 kV/cm), achieving 160%. The increase in foaming activity was due to the protein’s solubility and surface hydrophobicity, which enabled its movement to the bubble surface and the formation of a stable layer at the air–liquid interface. The application of PEF treatment may lead to the unfolding of the BSG protein, thereby exposing previously buried hydrophobic regions and reducing particle size. The modifications resulted in flexible protein structures with exposed hydrophobic regions, thereby improving their ability to adhere to interfaces and enhancing overall foaming efficacy [47,48].

#### 3.3.3. Emulsifying Properties

The ability of proteins to adsorb onto the surface of oil droplets, thereby stabilizing the oil–water interface and enhancing the resistance of the resulting emulsions to phase separation, is typically evaluated using the EA and ES [49,50]. Table 5 shows the EA and ES results for BSG proteins. PEF-treated BSG protein demonstrated a significantly higher EA (99.58%) than ALK-treated BSG protein (96.69%). The significantly increased hydrophobic–hydrophilic balance (HB/HL) and concentration of amphipathic amino acids, such tyrosine and methionine, in PEF-treated protein samples by approximately 14.86% and 25.89%, respectively (*p* < 0.05), may lead to enhanced emulsification.

This finding aligns with the study by [51], which found that applying PEF (10 kV/cm, 600 Hz, 3 min) to extract pea protein could enhance their emulsifying activity by up to 2-fold compared to the untreated sample. Ref. [39] demonstrated that enhanced EA and ES capabilities occurred when canola protein was extracted at increased field strengths, specifically from 10 kV to 30 kV. PEF may cause the exposure of hydrophobic regions in protein structures in these situations, including our study, which would improve the hydrophobic–hydrophilic balance in protein chains. This would increase the interactions between hydrophobic amino acids and oil through hydrophobic interactions and between hydrophilic amino acids and water, creating a barrier around oil droplets that would prevent aggregation and improve the stability of the emulsion overall [52,53]. This finding was in line with the foaming characteristics, which might be explained by higher surface hydrophobicity.

#### 3.3.4. Oil-Holding Capacity

The OHCs of the BSG protein concentrate produced by ALK and PEF were identical, ranging from 1.87 to 1.88 g/g of sample. Ref. [54] reported similar results, revealing that the OHC of wheat gluten concentrate subjected to PEF with 20,000 and 60,000 pulses was indistinguishable from the untreated sample. Ref. [55] indicated that PEF-treated wheat gluten protein did not alter OHC compared to the untreated sample, which ranged from 0.88 to 0.95 g/g sample. The oil-holding capacity method primarily relies on the physical trapping of oil within protein molecules. The PEF condition utilized in this study may mostly induce structural alterations in proteins rather than size reduction, resulting in an insignificant oil entrapment capacity compared to ALK-treated protein. However, the application of PEF treatment to proteins with varying amino acid profiles, particularly those rich in hydrophobic amino acids, may significantly affect oil-holding capacity.

## 4. Conclusions

PEF-assisted extraction is a preferable method for recovering high-quality protein from brewer’s spent grain. Compared to traditional alkaline extraction, PEF enhances protein yield, purity, and functional properties while minimizing structural damage. Furthermore, PEF positively influences the digestibility of protein, making it advantageous for incorporation into food products for the elderly. This sustainable approach can help upcycle BSG into valuable protein sources for food ingredients and other industrial applications. Future potential research could investigate the impact of brewer’s spent grains from various sources as well as the possibility of the combined application of pulsed electric fields and other extraction techniques on protein extraction and quality. In addition, the existing batch production process may hinder its commercial feasibility, suggesting that the advancement of a continuous PEF process for extraction purposes may also be of interest.

## Figures and Tables

**Table 1:** Protein purity and recovery obtained from alkaline and PEF-assisted extraction.

No.	Experimental Design			Responses	
	Number of Pulse (Pulse)	Field Strength (kV/cm)	Frequency (Hz)	Protein Purity (%)	Protein Recovery (%)
1	5000	8	9	85.28	15.58
2	9000	8	9	78.83	13.40
3	5000	10	9	91.60	21.64
4	9000	10	9	75.89	9.21
5	5000	9	8	90.87	25.48
6	9000	9	8	75.62	16.14
7	5000	9	10	89.96	8.75
8	9000	9	10	77.61	12.47
9	7000	8	8	77.48	9.32
10	7000	10	8	81.14	7.63
11	7000	8	10	80.80	15.86
12	7000	10	10	81.87	3.54
13	7000	9	9	77.60	13.14
14	7000	9	9	78.33	11.00
15	7000	9	9	76.98	11.68
Predicted value	5385.84	9.80	10.21	90.49	22.70
Experimental value	5386	10	10	89.26	23.72
ALK extraction (pH 10, stirring 90 min at 50 °C)				73.00	12.96

**Table 2:** ANOVA of the responses and model fit statistics for regression models.

Responses	Source	Sum of Square	DF	Mean Square	*F*-Value	*p*-Value
Protein purity	Model	420.17	9	46.69	39.28	0.0004
	X_1_	309.51	1	309.51	260.4	<0.0001
	X_2_	8.22	1	8.22	6.92	0.0465
	X_3_	3.29	1	3.29	2.77	0.1571
	X_1_^2^	66	1	66	55.53	0.0007
	X_2_^2^	3.96	1	3.96	3.33	0.1276
	X_3_^2^	10.06	1	10.06	8.46	0.0334
	X_1 × 2_	21.44	1	21.44	18.04	0.0081
	X_1 × 3_	2.1	1	2.1	1.77	0.241
	X_2 × 3_	1.68	1	1.68	1.41	0.2882
	Residual	5.94	5	1.19		
	Lack of Fit	0.2214				
	R^2^	0.9861				
	Adj R^2^	0.9609				
Protein recovery	Model	316.28	9	35.14	1.61	0.3122
	X_1_	51.24	1	51.24	2.35	0.1862
	X_2_	18.41	1	18.41	0.84	0.4007
	X_3_	40.32	1	40.32	1.85	0.2324
	X_1_^2^	85.71	1	85.71	3.92	0.1045
	X_2_^2^	12.03	1	12.03	0.55	0.4915
	X_3_^2^	4.07	1	4.07	0.19	0.6841
	X_1 × 2_	23.23	1	26.23	1.20	0.3231
	X_1 × 3_	42.64	1	42.64	1.95	0.2122
	X_2 × 3_	28.19	1	28.19	1.29	0.3074
	Residual	109.22	5	21.84		
	Lack of Fit	0.0327				
	R^2^	0.7433				
	Adj R^2^	0.2813				

**Table 3:** Chemical composition and secondary structure portions of proteins obtained from alkaline- and PEF-assisted extraction.

	ALK	PEF
Chemical composition (%)		
Moisture	4.41 ± 0.02 ^a^	3.72 ± 0.03 ^b^
Protein	73.00 ± 0.46 ^b^	89.26 ± 0.93 ^a^
Fat	1.90 ± 0.02 ^a^	1.10 ± 0.04 ^b^
Ash	2.31 ± 0.05 ^a^	1.02 ± 0.01 ^b^
Fiber	2.34 ± 0.55 ^a^	1.06 ± 0.06 ^b^
Carbohydrate	16.04 ± 0.88 ^a^	3.84 ± 0.12 ^b^
Portion of secondary structures (%)		
α-helices	22.95 ± 0.13 ^a^	22.18 ± 0.20 ^b^
β-sheets	22.15 ± 0.18 ^b^	23.01 ± 0.13 ^a^
β-turns	21.14 ± 0.51 ^a^	20.25 ± 0.23 ^b^
Random coils	33.76 ± 0.25 ^ns^	34.56 ± 0.29 ^ns^

Data are expressed as mean ± SD. Different lowercase letters (a, b) in the same row indicate significant differences (*p*< 0.05).

**Table 4:** The essential amino acid, amino acid scores, and PDCAASs of proteins obtained from alkaline- and PEF-assisted extraction.

Amino Acids	Amount of Essential Amino Acid (mg/100 g Sample)		Amino Acid Score **	
	ALK	PEF	ALK	PEF
Essential amino acids				
Histidine	1556.05 ± 0.90	2159.31 ± 10.59	1.42 ± 0.00	1.97 ± 0.01
Isoleucine	1150.96 ± 17.49	1456.10 ± 350.66	0.53 ± 0.01 *	0.66 ± 0.16 *
Leucine	1595.54 ± 14.70	2103.90 ± 2.44	0.37 ± 0.00 *	0.49 ± 0.00 *
Lysine	2046.99 ± 9.32	2697.46 ± 11.79	0.63 ± 0.00 *	0.82 ± 0.00 *
Methionine	991.32 ± 2.42	1293.33 ± 41.83	0.62 ± 0.00 *	0.81 ± 0.03 *
Phenylalanine	2896.92 ± 4.79	3108.10 ± 29.11	1.05 ± 0.00	1.31 ± 0.00
Threonine	2198.47 ± 17.95	3099.73 ± 4.43	1.32 ± 0.01	1.85 ± 0.00
Valine	2856.95 ± 6.11	3844.27 ± 15.25	1.01 ± 0.00	1.35 ± 0.01
Total	15,293.20 ± 71.14	20,288.28 ± 447.24	-	-
In vitro digestibility (%)	-	-	49.84 ± 0.10 ^b^	54.96 ± 0.48 ^a^
PDCAAS (%)	-	-	18.44 ± 0.11 ^b^ (Leucine)	24.42 ± 0.02 ^a^ (Leucine)

Data are expressed as mean ± SD. Different lowercase letters (a, b) in the same row indicate significant differences (*p* < 0.05). * Limiting amino acid. ** The AAS is calculated according to the body’s amino acid requirements for individuals over 18 years old.

**Table 5:** Properties of proteins obtained from alkaline- and PEF-assisted extraction.

Protein Properties	ALK	PEF
HB/HL balance	0.63 ± 0.00 ^b^	0.74 ± 0.01 ^a^
Amphipathic amino acids (mg/100 g)	3127.10 ± 17.12 ^b^	4219.55 ± 73.30 ^a^
Foaming properties		
Foamability (%)	7.84 ± 0.51 ^b^	30.86 ± 0.24 ^a^
Foam stability (%)	68.89 ± 1.92 ^b^	79.37 ± 5.02 ^a^
Emulsifying properties		
Emulsifying ability (%)	96.69 ± 1.41 ^b^	99.58 ± 0.72 ^a^
Emulsion stability (%)	90.98 ± 2.26 ^ns^	91.20 ± 2.25 ^ns^
OHC (g/g sample)	1.88 ± 0.09 ^ns^	1.87 ± 0.06 ^ns^

Data are expressed as mean ± SD. Different lowercase letters (a, b) in the same row indicate significant differences (*p* < 0.05), whereas “ns” indicates not significant (*p* > 0.05). HB/HL balance represents the ratio of hydrophobic amino acids to hydrophilic amino acids. Amphipathic amino acids include tyrosine and methionine.

## Data Availability

The original contributions presented in this study are included in the article. Further inquiries can be directed to the corresponding author.
